# A clinical severity scoring system for visceral leishmaniasis in immunocompetent patients in South Sudan

**DOI:** 10.1371/journal.pntd.0005921

**Published:** 2017-10-02

**Authors:** Suzette S. Kämink, Simon M. Collin, Tim Harrison, Francis Gatluak, Abdul Wasay Mullahzada, Koert Ritmeijer

**Affiliations:** 1 Public Health Department, Médecins Sans Frontières, Amsterdam, The Netherlands; 2 Department Health Sciences, Vrije Universiteit, Amsterdam, The Netherlands; 3 Population Health Sciences, Bristol Medical School University of Bristol, Bristol, United Kingdom; 4 Médecins Sans Frontières, Lankien, South Sudan; London School of Hygiene and Tropical Medicine, UNITED KINGDOM

## Abstract

**Background:**

South Sudan is one of the most endemic countries for visceral leishmaniasis (VL), and is frequently affected by large epidemics. In resource-limited settings, clinicians require a simple clinical tool to identify VL patients who are at increased risk of dying, and who need specialised treatment with liposomal amphotericin B and other supportive care. The aim of this study was to develop and validate a clinical severity scoring system based on risk factors for death in VL patients in South Sudan.

**Methods:**

A retrospective analysis was conducted of data from a cohort of 6,633 VL patients who were treated in the Médecins Sans Frontières (MSF) hospital in Lankien between July 2013 and June 2015. Risk factors for death during treatment were identified using multivariable logistic regression models, and the regression coefficients were used to develop a severity scoring system. Sensitivity and specificity of score cut-offs were assessed by receiver operating characteristic (ROC) analysis.

**Results:**

In multivariable models, risk factors for death in adult VL patients were: anaemia (odds ratio (OR) 4.46 (95% CI 1.58–12.6) for Hb <6g/dL compared with ≥9g/dL), nutritional status (OR 4.84 (2.09–11.2) for BMI <13 kg/m^2^ compared with ≥16 kg/m^2^), weakness (OR 4.20 (1.82–9.73) for collapsed compared with normal weakness), jaundice (OR 3.41 (1.17–9.95)), and oedema/ascites (OR 4.86 (1.67–14.1)). For children and adolescents the risk factors were: age (OR 10.7 (6.3–18.3) for age <2 years compared with 6–18 years), anaemia (OR 7.76 (4.15–14.5) for Hb <6g/dL compared with ≥9g/dL), weakness (OR 3.13 (22.8–105.2) for collapsed compared with normal weakness), and jaundice (OR 12.8 (4.06–40.2)). Severity scoring predictive ability was 74.4% in adults and 83.4% in children and adolescents.

**Conclusion:**

Our evidenced-based severity scoring system demonstrated sufficient predictive ability to be operationalised as a clinical tool for rational allocation of treatment to VL patients at MSF centres in South Sudan.

## Author summary

Visceral leishmaniasis (VL), also known as kala-azar, is a neglected tropical disease caused by a parasite (*Leishmania*) and transmitted to humans through the bite of a sandfly. South Sudan is one of the highest endemic countries for VL, frequently affected by epidemics. In South Sudan are different treatment options for VL available: the standard therapy given on ambulatory bases by intramuscular injections for 17 days, and specialized treatment for patients with severe VL, administered intravenously in a hospital over 12 days. In the extremely resource limited context of South Sudan, the most optimal treatment to patients with severe VL should be provided, but a rational use of drugs maintained. In this study, we identified risk factors for death in VL patients of South Sudan, and based on these risk factors we developed a severity scoring system. This severity scoring system will be a clinical decision making tool for allocation of VL patients to the appropriate treatment and to minimise the mortality of the VL patients in South Sudan.

## Introduction

Visceral leishmaniasis (VL, or kala-azar) is a neglected tropical disease which is caused by the obligate intracellular protozoa of the *Leishmania donovani* complex (*L*. *donovani* and *L*. *infantum*) (1). In East Africa VL is caused by *L*. *donovani*, and is transmitted anthroponotically by phlebotomine sandfly vectors [1].

VL targets the lymphatic and reticuloendothelial system, affecting spleen, liver, mucosa of the small intestine and respiratory tract, bone marrow, lymph nodes and the other lymphoid tissues, causing persistent fever, organomegaly, pancytopenia and wasting. Patients with VL are severely immunocompromised, and death occurs from opportunistic or concomitant infections, or from complications such as malnutrition, anaemia or bleeding. Without treatment VL is typically fatal [1, 2].

South Sudan is one of the most endemic countries for VL, with an annual incidence of 7,400–14,200 cases [3]. The country has been affected by frequent large epidemics, often associated with mass displacement due to armed conflict, and causing high mortality [4, 5].

Current first line treatment for VL in South Sudan is the pentavalent antimonial, sodium stibogluconate (SSG), in combination with an aminoglycoside, paromomycin (PM). SSG/PM is given on an ambulatory basis over 17 days with daily intramuscular injections [6, 7]. However, SSG is often poorly tolerated, and toxicity results in a significant incidence of serious adverse events such as pancreatic, hepato- and nephrotoxicity, cardiotoxicity, gastro-intestinal disorders and, in pregnant women, spontaneous abortion [8–11]. For this reason, SSG is contraindicated for specific patient groups (e.g. pregnant women or HIV co-infected) or in patients with severe VL. These patients should be treated with liposomal amphotericin B (AmBisome) [12]. AmBisome is much better tolerated but is much more expensive and requires cold chain transportation, cool storage, intravenous administration, and hospitalisation for at least 12 days.

These are major challenges in the resource limited context of South Sudan, meaning that rational use of AmBisome is currently a key operational requirement. AmBisome needs to be reserved for severely ill VL patients who are at high risk of dying or at risk of SSG intolerability [12–14], whilst standard SSG/PM treatment can continue to be administered to patients with uncomplicated VL [7]. In resource-limited settings, clinicians require a simple clinical tool to identify VL patients who are at increased risk of dying, and who need specialised treatment with liposomal amphotericin B and other supportive care.

The aim of this study was to develop an evidenced-based risk scoring system which could be used as a clinical decision making tool in the field, to help clinicians decide whether a VL patient requires intensive VL care and treatment with AmBisome or standard VL management and less intensive monitoring. The risk scoring system would be based on risk factors for death during treatment in a retrospective cohort of VL patients, and would be validated internally and against older cohorts of VL patients treated by MSF in South Sudan.

## Methods

### Ethics statement

This research fulfilled the exemption criteria set by the Médecins Sans Frontières Ethical Review Board for a posteriori analyses of routinely collected clinical data, and thus did not require MSF ERB review. It was conducted with permission from the Medical Director of the MSF Operational Centre Amsterdam. All data were anonymised before analysis.

### Study design and data source

A retrospective analysis was conducted of routinely collected data from a cohort of 6,821 VL patients who attended the hospital of Médecins Sans Frontières (MSF) in Lankien, Jonglei state, South Sudan during the VL outbreak between July 2013 and June 2015. The data were stored in an electronic database, and included key dates, demographic, anthropometric, diagnostic and clinical characteristics of patients, treatment regime and outcome. Patients with incomplete data and who defaulted from treatment were excluded from analyses. VL-HIV coinfected patients were excluded because treatments and outcomes are different in this small immunocompromised subgroup (during the study period the HIV/VL rate in Lankien was only 0.15%).

### Outcome

Our analysis was based on a binary outcome of died (during or immediately after treatment) or survived treatment. “Survived” means that the patient was discharged after successful clinical response to treatment: absence of fever, reduction of spleen and liver size, increased haemoglobin, restored appetite, and feeling well. Patients with an increased risk of treatment failure or relapse (i.e. patients with a prior episode of VL, or patients with inadequate or doubtful clinical response) require a negative parasitological test-of-cure by aspirate microscopy to confirm cure of VL.

There is no systematic follow up of patients after discharge, because they return to areas remote from the treatment centre. A defaulter was defined as a patient who did not complete treatment, and had an unknown outcome.

### Risk factors

The dataset included the following potential risk factors for death in VL: age (years), sex, presence/absence of jaundice, lymphadenopathy, oedema/ascites; prior episode/relapse of VL, Hb level (g/dL), spleen size (cm below the left costal margin), self-reported duration of illness (months), and nutritional status (body mass index (BMI) in patients ≥19years (kg/m^2^) and weight for height Z scores (WHZ) in patients <19 years old). State of weakness, was defined as ‘normal weakness’ (non-severe); ‘severe weakness’ (if patient needs assistance in walking or, in babies, if unable to sit up); or ‘collapse’ (if patient is unable to sit or drink or, in babies, if hypotonic and unable drink unaided). All of these variables have been identified as risk factors for death in earlier VL patient cohorts from South Sudan [2, 5, 15, 16].

### Diagnosis

All patients presenting with a history of fever more than 2 weeks and splenomegaly and/or lymphadenopathy and/or wasting (BMI <16 mg/m2 or <-2 Zscore) were considered VL suspects and underwent further diagnostic evaluations. Patients without prior VL treatment history (suspect primary VL) were first screened using the rK39 rapid diagnostic test (IT-Leish, Bio-Rad laboratories, USA) and a positive result confirmed VL. Those testing negative were screened with the leishmania direct agglutination test (DAT, Royal Tropical Institute, Amsterdam, The Netherlands) and a high titer (≥1:6400) confirmed VL. Those with an intermediate DAT titer (1:800–1:3200) underwent tissue aspiration (spleen or lymph node) and positive result confirmed VL. Patients with prior VL treatment history (suspect relapse VL) underwent tissue aspiration and a positive result confirmed VL. A clinical diagnosis was made in patients contra-indicated for spleen aspirate (i.e. severely anaemic, bleeding, pregnant or collapsed) and didn’t have palpable lymph nodes or those with negative lymph node aspirate results but with persistent strong VL clinical suspicion in the absence of differential diagnoses [17].

### Treatment

The first line treatment was with SSG (20 mg/kg) in combination with PM (15 mg/kg) given on an ambulatory basis over 17 days with daily intramuscular injections.

Specific patients groups with contra-indication for SSG (pregnant women, HIV co-infected) or patients with known poor tolerability of SSG (age >45 years, severe VL) were treated with AmBisome by 6 IV infusions of 5 mg/kg on alternate days. Treatment had started without delay on the same day as confirmation of diagnosis.

If clinical and laboratory investigations confirm severe VL disease, specialised treatment was started immediately. ‘Specialised treatment’ means besides AmBisome also rehydration, aggressive antibiotic treatment (ceftriaxone IV), other supportive treatment (e.g. for malnutrition), intensified monitoring and treatment of any suspected abnormality.

As the aim is to predict a patient’s prognosis at the time of diagnosis, the treatment was not included in the analysis.

### Statistical analysis

#### Risk factors

Univariate and multivariable logistic regression models were used to quantify the associations of risk factors with death as odds ratios (ORs) with 95% confidence intervals (CIs). Two prediction models were built: one with patients aged 19 years and older (adults), and one with the patients younger than 19 years (defined as children and adolescents). Variables were included in the multivariable analysis if they had a significance level of p <0.2 in the univariate analysis, and backwards elimination was then used to build final prediction models.

#### Derivation of the severity scoring system

The severity scoring system was developed using a method described by Barquet et al, which is based on the Spiegelhalter-Knills Jones method [18–20]. Regression coefficients (RCs) from the final multivariable prediction model were used to calculate a score for each level of each risk factor (by dividing each RC by the smallest RC in the model and rounding up or down to the nearest integer). The reference level of each categorical variable was scored as ‘0’. The severity score for an individual patient is the sum of his/her severity scores for each risk factor; a higher score indicating higher risk of death, i.e. more severe illness [18, 21]. Probabilities of death can be calculated for each severity score, but the rounding up or down of each RC means that combinations of different levels of risk factors can lead to the same severity score, i.e. for each severity score there is a range of probabilities of death. The upper and lower limits of this range for each severity score was calculated using the method described by Coura-Vital et al [22].

#### Discriminative ability of the risk scoring system

The discriminative (predictive) ability of the severity scoring system was assessed using receiver operating characteristic (ROC) analysis [25, 26]. Discriminative ability was quantified by area under the curve (AUC), categorised as: ‘no better than a random guess’ (AUC 0.5–0.6); ‘poor’ (AUC 0.6–0.7); ‘fair’ (AUC 0.7–0.8); ‘good’ (AUC 0.8–0.9) or ‘excellent’ (AUC 0.9–1.0) [23, 24].

#### Determination of severity score thresholds

ROC analysis was used to calculate the sensitivity and specificity of different risk score thresholds [25, 26]. The optimum threshold can be determined by considering the clinical and operational implications of the sensitivity and specificity of different thresholds.

#### Validation

The severity scoring system was validated in three datasets from previous studies in South Sudan: two from patients treated in Lankien during 1999–2002 (N = 708) and during 2002–2005 (N = 1882), and one from patients treated in Malakal during 2002–2005 (N = 1757) [2, 15]. Validation was performed with the use of the Z test by comparing the predictive ability (AUC) of the severity scoring system across the datasets [27].

#### Statistical software

SPSS for Windows 23.0 and STATA/IC 14.0 were used for the statistical analyses.

## Results

### Study population

The initial dataset included 6,821 VL patients. After excluding patients with incomplete data (n = 8), HIV co-infection (n = 11), who defaulted from treatment (n = 159) or referred to another non MSF facility (n = 10) the total sample size was 6,633. Of these, 3,631 (54.7%) were male and 3,002 (45.3%) were female. Out of the 6,614 patients of whom the duration of illness was known 6,087 (92%) presented within 1 month after onset of symptoms, and no patient presented later than 6 months.

Of the 6,615 patients whose treatment regime was known, 5,149 patients were treated with SSG/PM and 1,466 patients with AmBisome. The data comprised 4,931 (74.3%) children and adolescents (aged < 19 years) and 1,702 (25.7%) adults (≥19 years). Mortality data was captured during the complete time of admission in the hospital, until discharge or death; the longest admission duration was 134 days. In total 6,447 patients (97.7%) survived and 186 (2.8%) died during treatment; 33% (49/186) of the deaths occurred within 48 hours of admission.

The characteristics of patients in each age group who died compared with those who survived are shown in Table 1. Mortality in children and adolescents was 2.4% compared with 4.1% among adults (OR 1.78 (95% CI 1.32–2.41)).

**Table 1:** Characteristics of the study population by outcome (survived or died).

	Adults (patients ≥19 years old)			Ch Children and Adolescents (patients <19 years old)		
Variable		Died n = 70 (4.1%)	Survived n = 1632 (95.9%)		Died n = 116 (2.4%)	Survived n = 4815 (97.5%)
**Age, years**						
	18–25	11 (2.2)	493 (97.8)	6–18	22 (0.7)	2949 (99.2)
	26–35	20 (3.2)	602 (96.8)	2–5	37 (2.5)	1424 (97.5)
	36–45	17 (6.4)	247 (93.6)	< 2	57 (11.4)	442 (88.6)
	> 45	22 (7.1)	290 (92.9)			
**Nutrition status**	**BMI, kg/m^2^**			**WHZ**		
	≥ 16	14 (1.7)	806 (98.3)	>-2	27 (2.3)	1137 (97.7)
	14.5–15.9	25 (5.1)	468 (94.9)	≤-2	35 (2.1)	1619 (97.9)
	13–14.4	18 (6.5)	259 (93.5)	≤-3	32 (1.3)	1652 (98.7)
	< 13	13 (11.6)	99 (88.4)	≤-4	21 (6.1)	323 (93.9)
**Sex**						
	male	42 (4.4)	915 (95.6)	male	59 (2.2)	2615 (97.8)
	female	28 (3.9)	717 (96.1)	female	57 (2.5)	2200 (97.5)
**State of weakness**						
	normal weakness	36 (2.6)	1345 (97.4)	normal weakness	83 (1.8)	4563 (98.2)
	severe weakness	24 (9.1)	239 (90.9)	severe weakness	28 (10.5)	239 (89.5)
	collapse	10 (17.2)	48 (82.8)	collapse	5 (41.7)	7 (58.3)
**Hb, g/Dl**						
	≥ 9	37 (3.1)	1174 (96.9)	≥ 9	25 (1.0)	2562 (99.0)
	7.5–8.9	17 (5.2)	310 (94.8)	7.5–8.9	29 (2.1)	1352 (97.9)
	6–7.4	10 (7.4)	126 (92.6)	6–7.4	34 (4.3)	749 (95.7)
	< 6	6(21.4)	22 (79.6)	< 6	28 (15.6)	151 (84.3)
**Duration of illness, months**						
	= 1	49 (3.9)	1195 (96.1)	= 1	88 (2.3)	3714 (97.7)
	< 1	12 (4.3)	268 (95.7)	< 1	24 (3.2)	737 (96.8)
	≥ 2	9 (5.2)	164 (94.8)	≥ 2	**4** (1.1)	350 (98.9)
**Jaundice**	no	64 (3.8)	1613 (96.2)	no	111 (2.3)	4790 (97.7)
	yes	6 (24.0)	19 (76.0)	yes	5 (17.9)	23 (82.1)
**Oedema/ascites**	no	64 (3.8)	1610 (96.2)	no	110 (2.3)	4766 (97.8)
	yes	6 (21.4)	22 (79.6)	yes	6 (10.9)	49 (89.1)
**Lymphadenopathy**	no	20 (5.6)	337 (94.4)	no	37 (4.0)	898 (96.0)
	yes	50 (3.7)	1290 (96.3)	yes	79 (2.0)	3907 (98.0)
**Prior episode VL**	0	61 (4.0)	1475 (96.0)	0	99 (2.2)	4347 (97.8)
	1 or more	9 (5.4)	157 (94.6)	1 or more	17 (3.5)	468 (96.5)
**Spleen size, cm**						
	< 1	31 (4.9)	596 (95.1)	< 1	40 (2.7)	1459 (97.3)
	1–4	21 (4.0)	502 (96.0)	1–4	50 (2.5)	1936 (97.5)
	5–7	10 (3.7)	262 (96.3)	5–7	15 (1.8)	803 (98.2)
	>7	8 (2.9)	268 (97.1)	>7	11 (1.8)	609 (98.2)

### Risk factors in adults

Univariate analysis showed that age, Hb, state of weakness, nutritional status, jaundice, and oedema/ascites were strongly associated with VL mortality (Table 2): patients >45 years old had 3.4-fold higher odds of death (OR 3.42 (95% CI 1.64–7.16)) compared with patients aged 18–25 years; patients in a state of collapse were 8 times more likely to die (OR 7.80 (95% CI 3.66–16.6)) compared with patients who arrived in a ‘normal’ state of weakness; Hb levels <6g/dL increased the odds of dying almost 9-fold (OR 8.67 (95% CI 3.32–22.6)) compared with levels ≥9g/dL; and BMI <13 kg/m^2^ was associated with 7.6-fold higher odds of death (OR 7.56 (95% CI 3.45–16.6)) compared with BMI ≥ 16 kg/m^2^. Presence of jaundice and oedema/ascites increased the odds of dying 8-fold (OR 7.95 (95% CI 3.07–20.6)) and almost 7-fold (OR 6.87 (95% CI 2.69–17.5)), respectively. Sex, lymphadenopathy, prior episode VL, duration of illness, and spleen size were not associated with risk of death.

**Table 2:** Risk factors for death among adult patients (≥19 years old).

Variable (n = 1,702)	Category	Univariate logistic regression		Final prediction model		
		Crude OR^a^ (95% CI^b^)	P-value^c^	RC^d^	Adjusted OR (95%CI)	P–value^c^
**Sex**	male		0.52			
	female	0.85(0.52–1.39)				
**Age, years**	18–25		0.002			
	26–35	1.50 (0.71–3.15)				
	36–45	3.10 (1.43–6.73)				
	> 45	3.42 (1.64–7.16)				
**State of weakness**	normal weakness		< 0.001			
	severe weakness	3.76 (2.20–6.41)		0.90	2.45 (1.39–4.33)	0.002
	collapse	7.80 (3.66–16.6)		1.44	4.20 (1.82–9.73)	0.001
**BMI, kg/m^2^**	≥ 16		< 0.001			
	14.5–15.9	3.08 (1.58–5.98)		0.96	2.60 (1.32–5.14)	0.006
	13–14.4	4.00 (1.96–8.16)		1.02	2.78 (1.31–5.91)	0.008
	< 13	7.56 (3.45–16.6)		1.58	4.84 (2.09–11.2)	< 0.001
**Hb, g/dL**	≥ 9		< 0.001			
	7.5–8.9	1.74 (0.97–3.14)		0.07	1.08 (0.57–2.03)	0.82
	6–7.4	2.52 (1.23–5.19)		0.42	1.52 (0.70–3.30)	0.29
	< 6	8.67 (3.32–22.6)		1.50	4.46 (1.58–12.6)	0.005
**Oedema/ascites**	no		< 0.001			0.004
	yes	6.87 (2.69–17.5)		1.58	4.86 (1.67–14.1)	
**Jaundice**	no		< 0.001			0.025
	yes	7.95(3.07–20.6)		1.23	3.41 (1.17–9.95)	
**Lymphadenopathy**	no		0.12			
	yes	0.65(0.38–1.11)				
**Prior episode VL**	0		0.37			
	1 or more	1.39 (0.68–2.85)				
**Duration of illness, months**	= 1		0.73			
	< 1	1.09 (0.57–2.08)				
	≥ 2	1.34 (0.65–2.78)				
**Spleen size, cm**	< 1		0.53			
	1–4	0.80 (0.46–1.42)				
	5–7	0.73 (0.35–1.52)				
	>7	0.57 (0.26–1.27)				

^a^ OR indicates odds ratio

^b^ CI indicates confidence interval

^c^ P value from Chi squared test

^d^ RC indicates (unexponentiated) regression coefficient

The effects of Hb, state of weakness, nutritional status, jaundice, and oedema/ascites were reduced by mutual adjustment, and age was eliminated from the final prediction model (Table 2). In this model, patients in a state of collapse were 4 times more likely to die (OR 4.20 (95% CI 1.82–9.73)) compared with patients who arrived in a ‘normal’ state of weakness; Hb levels <6g/dL increased the odds of dying 4.5-fold (OR 4.46 (95% CI 1.58–12.6)) compared with levels ≥9g/dL; and BMI <13 kg/m^2^ was associated with almost 5-fold higher odds of death (OR 4.84 (95% CI 2.09–11.2)) compared with BMI ≥ 16 kg/m^2^. Presence of jaundice and oedema/ascites increased the odds of dying >3-fold (OR 3.41 (95% CI 1.17–9.95)) and almost 5-fold (OR 4.86 (95% CI 1.67–14.1)), respectively.

### Risk factors in children and adolescent

The univariate analyses of children and adolescents showed that age, Hb, state of weakness, jaundice, oedema/ascites and WHZ were strongly associated with VL mortality (Table 3).

**Table 3:** Risk factors for death among child and adolescent patients (<19 years old).

		Univariate logistic regression		Final prediction model		
Variable (n = 4,931)	Category	Crude OR^a^ (95% CI^b^)	P^c^ value	RC^d^	Adjusted OR (95%CI)	P value^c^
**Sex**	male		0.46			
	female	1.15 (0.79–1.66)				
**Age, years**	6–18		< 0.001			
	2–5	3.48 (2.05–5.93)		0.98	2.67 (1.55–4.61)	< 0.001
	<2	17.29 (10.46–28.6)		2.37	10.74 (6.31–18.3)	< 0.001
**State of weakness**	normal weakness		< 0.001			
	severe weakness	6.44 (4.12–10.1)		0.92	2.53 (1.53–4.18)	< 0.001
	state of collapse	39.27(12.21–126.3)		3.13	22.82 (4.95–105.2)	< 0.001
**WHZ**	> -2		< 0.001			
	= -2	0.91 (0.55–1.51)				
	= -3	0.87 (0.49–1.37)				
	= -4	2.74 (1.58–4.90)				
**Hb, g/dL**	≥ 9		< 0.001			
	7.5–8.9	2.20 (1.28–3.77)		0.57	1.77 (1.02–3.08)	0.044
	6–7.4	4.65 (2.76–7.85)		1.02	2.77 (1.59–4.82)	< 0.001
	< 6	19.00 (10.81–33.4)		2.05	7.76 (4.15–14.5)	< 0.001
**Oedema/ascites**	no		<0.001			
	yes	5.31 (2.25–12.7)				
**Jaundice**	no		< 0.001			< 0.001
	yes	9.38 (3.50–25.2)		2.55	12.77 (4.06–40.2)	
**Lymphadenopathy**	no		< 0.001			
	yes	0.49 (0.33–0.73)				
**Prior episode VL**	0		0.08			
	1 or more	1.60 (0.94–2.69)				
**Duration of illness, months**	= 1		0.12			
	< 1	1.37 (0.87–2.17)				
	≥ 2	0.48 (0.18–1.32)				
**Spleen size, cm**	< 1		0.44			
	1–4	0.94 (0.17–1.44)				
	5–7	0.68 (0.37–1.24)				
	>7	0.66 (0.34–1.29)				

^a^ OR indicates odds ratio

^b^ CI indicates confidence interval

^c^ P value from Chi squared test

^d^ RC indicates (unexponentiated) regression coefficient

Patients < 2 years old had >17-fold higher odds of death (OR 17.3 (95% CI 10.5–28.6)) compared with patients aged 6–18 years; patients in a state of collapse were 39 times more likely to die (OR 39.3 (95% CI 12.2–126.3)) compared with patients who arrived in a ‘normal’ state of weakness; Hb levels <6g/dL increased the odds of dying 19-fold (OR 19.0 (95% CI 10.8–33.4)) compared with levels ≥9g/dL; and WHZ <-4 was associated with almost 3-fold higher odds of death (OR 2.74 (95% CI 1.58–4.90)) compared with WHZ > -2. Presence of jaundice and oedema/ascites increased the odds of dying >9-fold (OR 9.38 (95% CI 3.50–25.2)) and >5-fold (OR 5.31 (95% CI 2.25–12.7)), respectively.

For patients with lymphadenopathy, the odds of dying reduced by half. (OR 0.49 (95% CI 0.33–0.73) Sex, prior episode VL, and spleen size were not associated with risk of death. Duration of illness did not have sufficient cases for analyses.

The effects of age, Hb, state of weakness and jaundice were reduced by mutual adjustment, and lymphadenopathy, nutritional status and oedema/ascites were eliminated from the final prediction model (Table 3). In this model, patients < 2 years old had almost 11-fold higher odds of death (OR 10.7 (95% CI 6.31–18.26)) compared with patients aged 6–18 years; patients in a state of collapse were almost 23 times more likely to die (OR 22.8 (95% CI 4.95–105.2)) compared with patients who arrived in a ‘normal’ state of weakness; Hb levels <6g/dL increased the odds of dying nearly 8-fold (OR 7.76 (95% CI 4.15–14.5)) compared with levels ≥9g/dL; and presence of jaundice increased the odds of dying 3.4-fold (OR 3.41 (95% CI 1.17–9.95)).

### Severity scoring system

The scores for each variable in the final prediction model are shown in Table 4, and the range of probabilities for each score (due to rounding up or down of regression coefficients) are presented in Table 5. For adults, probability of death exceeded 10% for risk scores ≥3 (6.1% (104/1695) of adults); in children and adolescents, the threshold for exceeding a 10% probability of death was a score ≥6 (6.8% (339/4921) of children).

**Table 4:** Severity scoring for adults (≥ 19 years), and children and adolescents (< 19 years).

		Severity Score	
Variable	Category	Adults	Children and adolescents
**State of weakness**	normal weakness	0	0
	severe weakness	1	2
	state of collapse	2	5
**Hb, g/dL**	≥ 9.0	0	0
	7.5–8.9	0	1
	6.0–7.4	0	2
	< 6.0	2	4
**BMI, kg/m^2^**	≥ 16	0	
	14.5–15.9	1	
	13.0–14.4	1	
	< 13	2	
**Jaundice**	not present	0	0
	present	1	4
**Oedema/ascites**	not present	0	
	present	2	
**Age, years**	6–18		0
	2–5		2
	< 2		4

**Table 5:** Probabilities of death^a^ at each score for adults (≥ 19 years), and children and adolescents (< 19 years)^b^.

	Adults				Children and adolescents			
			Probability of death				Probability of death	
Severity score	All cases (n)	Observed case fatality (n)	Lower limit	Upper limit	All cases (n)	Observed case fatality (n)	Lower limit	Upper limit
**0**	719	10	1.2%	1.4%	1712	8	0.4%	0.5%
**1**	640	20	2.9%	4.0%	745	6	0.7%	0.7%
**2**	232	19	4.9%	10.3%	1001	9	1.0%	1.1%
**3**	77	13	11.8%	22.1%	450	6	1.8%	2.0%
**4**	21	6	18.6%	35.9%	506	13	2.7%	5.0%
**5**	6	2	37.3%	57.9%	168	17	4.8%	8.6%
**6**	-	-	52.5%	71.4%	219	26	7.2%	14.3%
**7–8**	-	-	74.2%	89.5%	95	20	17.8%	36.1%
**9–10**	-	-	94.8%	94.8%	19	7	37.5%	47.1%
**11–17**	-	-	-	-	6	4	46.1%	99.0%

^a^Low risk (green) <3% probability of death; Moderate risk (yellow) 3–10% probability of death; High risk (red) >10% probability of death.

^b^ The number of cases in this table (adults N = 1695, children/adolescents N = 4921) are slightly lower than shown in Table 1 (adults N = 1702, children/adolescents N = 4931) due to missing data for some of the predictive variables.

**Fig 1:**
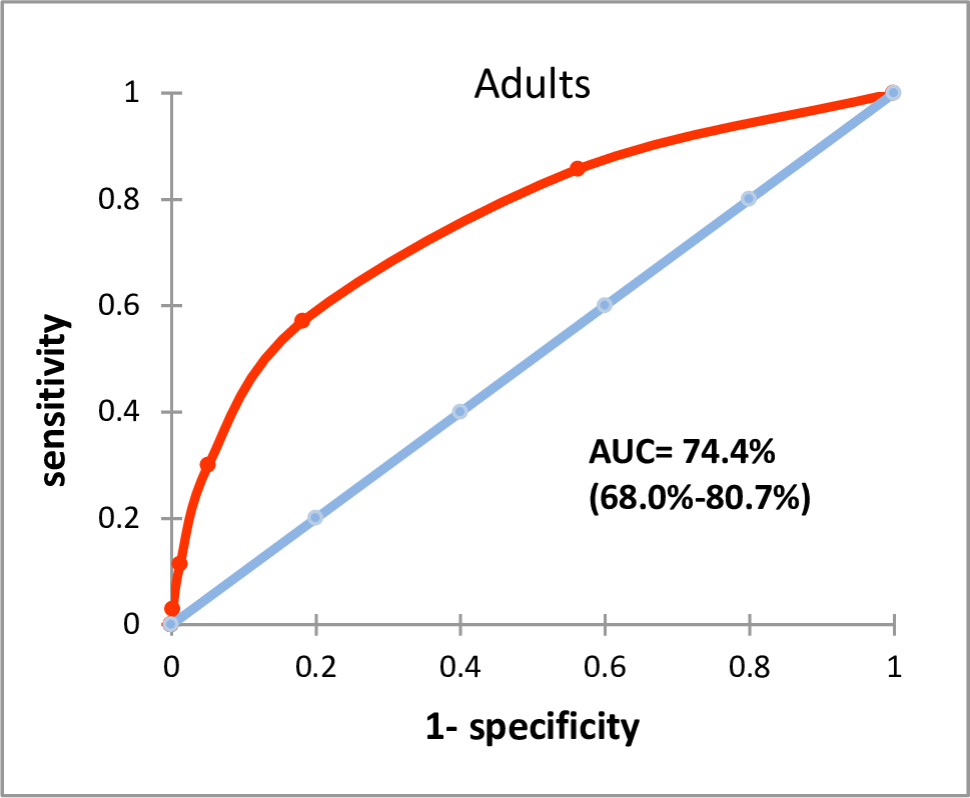
Area under the curve for adults scoring.

**Fig 2:**
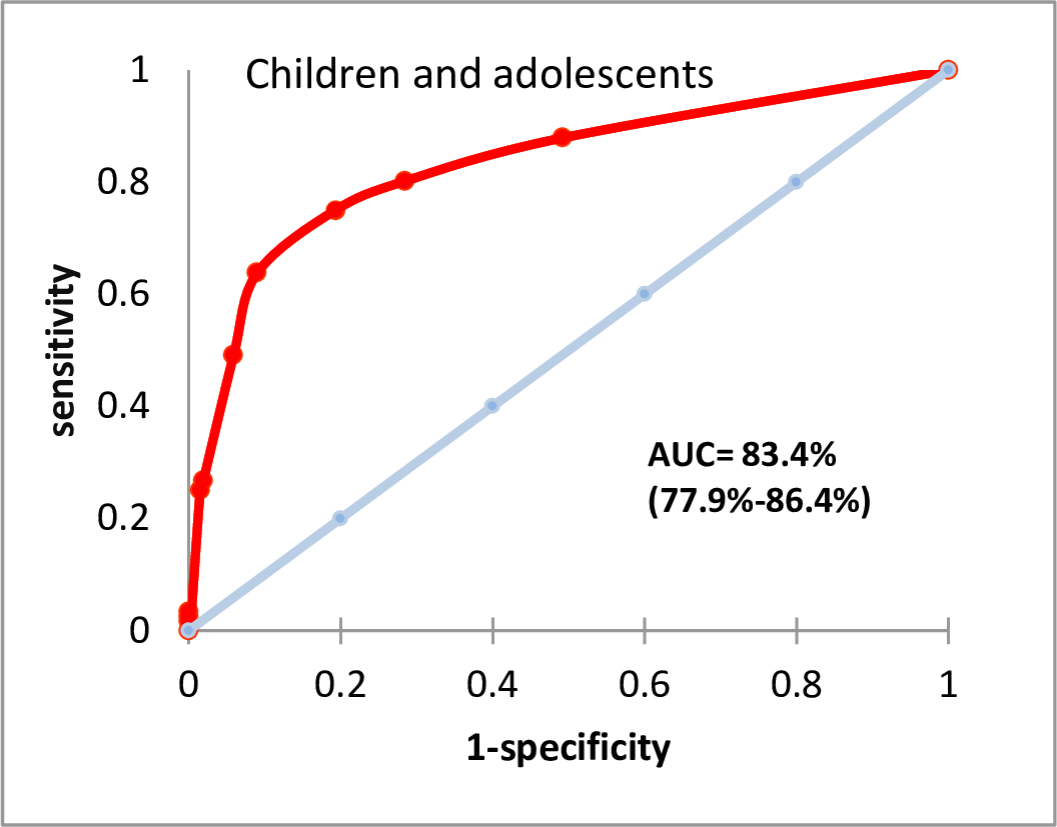
Area under the curve for children and adolescents scoring.

For the severity scoring of the adults the classification matrix resulted in 80.8% correctly predicted deaths. The AUC of the severity scores of the adults gave an overall predictive performance of 74.4% (95 CI 68.0%-81.0%), indicating ‘fair’ predictive ability (Fig 1). For children and adolescents, the classification matrix showed 80.6% correctly predicted. The AUC was 83.4% (95% CI 78.0%-86.8), interpreted as ‘good’ predictive ability (Fig 2). Sensitivity >55% required a score ≥2 in adults (sensitivity 57%, specificity 82%) and ≥5 in children and adolescents (sensitivity 64%, specificity 91%). Sensitivity >75% required a score ≥1 in adults (sensitivity 86%, specificity 44%) and ≥4 in children and adolescents (sensitivity 75%, specificity 81%) (Table 6).

**Table 6:** Performance of the severity scoring for prediction of death in VL patients in South Sudan.

Severity score	Adults		Children and adolescents	
	Sensitivity	Specificity	Sensitivity	Specificity
**0**	100%	0.0%	100%	0.0%
**1**	85.7%	43.7%	-	-
**2**	57.1%	81.8%	87.9%	50.8%
**3**	30.0%	94.9%	80.2%	71.5%
**4**	11.4%	98.8%	75.0%	80.7%
**5**	2.9%	99.8%	63.8%	91.0%
**6**	0.0%	100%	49.1%	94.1%
**7**	0.0%	100%	26.7%	98.1%
**8**	0.0%	100%	25.0%	98.5%
**9–10**	0.0%	100%	9.5%	99.8%
**11**	-	-	3.4%	100%
**12**	-	-	2.6%	100%
**13**	-	-	1.7%	100%
**14**	-	-	0.0%	100%

### Validation

External validation of the scoring for adults with the Lankien datasets of 1999–2002, 2002–2005 and the Malakal dataset of 2002–2005 yielded AUC of 72.2%, 79.5% and 71.2%, respectively. Discriminative ability did not differ significantly across the four datasets (p = 0.48). For children and adolescents, corresponding AUC were 72.2%, 82.8% and 76.6%, with only very weak evidence of a difference in discriminative ability across the three datasets (p = 0.13).

## Discussion

In this study, the risk factors for death in VL patients in South Sudan during the VL epidemic from 2013 to 2015 were analysed. Significant risk factors for adult patients were nutritional status (BMI), Hb, weakness status, jaundice and oedema/ascites. In children and adolescents, the risk factors were age, Hb, weakness status and jaundice. Using these risk factors, an evidence-based clinical severity scoring system was developed that may be able to determine reliably and easily a patient’s risk of dying, thereby enhancing the rational use of more costly and complex VL treatments. The overall accurate predictive ability of this severity scoring system was confirmed by external validation in other data from the same setting.

VL treatment effect was not analysed, because in observational studies indications for treatment are usually not standardised, and confounding by indication could lead to bias. Moreover, as the aim is to predict a patient’s prognosis at the time of diagnosis, the treatment should not be included.

In operationalising a risk scoring system, erring towards a higher sensitivity over a lower specificity would mean that >28% of child and > 56% of adult patients should receive specialised VL care. This is unfortunately not possible in a severely resource limited setting such as South Sudan. The optimal threshold will be a compromise between sensitivity and specificity, i.e. a threshold needs to be chosen that includes as many patients with increased risk of dying as possible, whilst maintaining a rational use of scarce resources. Whilst our focus has been to reduce VL mortality by identifying those patients most at risk of dying, the severity scoring system could be a useful tool in the management of patients at lower risk. VL patients with a low risk of dying (<3.0%), could be treated on an ambulatory basis in outpatient treatment centres. Patients with a moderate risk of dying (3–10%) could be treated in an outpatient department, but under close monitoring by experienced clinicians. Patients with a higher score and thus a higher risk of dying (>10%) should be admitted as inpatients for specialised VL care and intensive monitoring.

In this study there was no association of acute malnutrition (WHZ <-2) with death in children and adolescents, although severe acute malnutrition (WHZ <-4) was associated with increased risk of death. This finding is in contrast with earlier studies in South Sudan in 2004 and 1991 [15, 16]. The lack of association at less severe levels of malnutrition in our study could be explained by improved treatment of malnourished children in an intensive therapeutic feeding centre (ITFC), where malnourished children receive specialised medical and nutritional care. This specialized treatment may have mediated the impact of malnutrition on VL mortality in children, except for the very severely malnourished. In our final prediction model, severely malnourished children would score highly for being in a state of collapse and/or presenting with very low Hb levels, and would exceed the threshold for specialised care.

A finding which is in line with previous studies in South Sudan is that young age was a strong predictor for VL mortality [15, 16]. The eleven-fold higher odds of death in children below 2 years (compared with older children) demonstrate the vulnerability of this youngest age group. Accordingly, age <2 years contributed 4 points, and even without additional risk factors already is at ‘moderate risk’. On the other hand, older age (>45 years), which was strongly associated with mortality in the univariate analysis, was not retained as an independent risk factor in the multivariable regression model, contrary to earlier studies [15, 16, 22]. In these earlier studies, most elderly patients were treated with SSG, whereas in the present study 87% of the patients older than 45 years were treated with AmBisome [15, 16, 28]. Several studies had demonstrated a high mortality in older patients due to SSG toxicity, and during those earlier studies there was no or only limited AmBisome available [12]. Therefore in our dataset, AmBisome may have mediated the effect of older age on VL mortality. This seems to confirm the recommendation that treatment with AmBisome may be lifesaving for the elderly VL patients [9].

In contrast with two previous studies we did not find that splenomegaly was associated with increased risk of death [15, 29]. Conversely, we found a crude inverse association of lymphadenopathy with risk of death (although not evident when adjusted for other risk factors). We can think of no plausible explanation for these apparently anomalous findings.

The mortality rate in this study cohort was much lower than in earlier studies (2.8% vs. 10.9% and 10.0%) [7, 16]. Despite the fact that the difficult context of civil war, violence and displacement, the weak and unstable health care system was similar between this study and the earlier studies in South Sudan [30, 31]. This may be partially explained by earlier presentation at the Lankien hospital: more than 92% of the patients were able to present early, i.e. within one month after onset of clinical disease, whereas only 65% of the patient were able to during those earlier studies. As there were only 13 deaths in 527 patients presenting more than 1 month after onset of symptoms, duration of illness could not be identified as a risk factor. This seems to support the interpretation that specific joint efforts led since 2009 by the World Health Organisation in collaboration with Ministry of Health and nongovernmental organisations (NGOs), have been successful in achieving improved access to VL care in South Sudan (by decentralising VL treatment services and ensuring supply of tests and drugs) [3, 30].

The main strength of our study is that it was based on a large dataset with very few patients excluded because of missing data. Also, a robust external validation was possible, because of the availability of MSF datasets from previous VL outbreaks in South Sudan. The study was based on a cohort of South Sudanese patients, and therefore the predictors of death and the severity scoring system should not be generalised to patient populations in other countries. For example, where VL is caused by other Leishmania strains, where HIV co-infection is more prevalent, or where resistance to SSG is more common or AmBisome is more affordable. Another limitation is that this retrospective study was conducted with routinely collected programme data. Therefore it may be missing out on other important risk factors that were not included in this database, such as laboratory parameters (e.g. related to electrolyte disturbances, blood cell counts, or immune status). Although one third of deaths occurred within the first 48 hours after admission, which may limit the impact of our severity scoring system on mortality, the predictive ability and simplicity of the system means that it can be easily operationalised and implemented in the field.

The scoring system presented in this study only includes clinical parameters and a simple Hb lab test, and it therefore presents a practical tool that can be used in all field hospitals and health centres in the VL endemic areas in South Sudan. Clinicians will use their clinical judgement and experience to make treatment decisions, aided by the risk scoring tool. Given the strong associations between known risk factors and mortality, it would not be ethical to attempt a randomised trial of the severity scoring system, but we would hope to see a (continued) overall improvement in treatment outcomes in VL programmes in South Sudan until new safe, effective, and affordable treatment becomes available for all patients.

## Supporting information

S1 File STROBE checklist. (DOCX)

S1 Fig VL patient receives the painful SSG injection in Pieri, Jonglei state, South Sudan, 2013. Image credit: M.den Boer, MSF.
(TIF)
