# Supplementary material for: A clinical severity scoring system for visceral leishmaniasis in immunocompetent patients in South Sudan
Source: PLoS Negl Trop Dis. 2017 Oct 2;11(10):e0005921. doi: 10.1371/journal.pntd.0005921 (PMC5638606; doi:10.1371/journal.pntd.0005921)
Supplement: S1 File — (DOCX) [file pntd.0005921.s001.docx]

STROBE Statement—checklist of items that should be included in reports of observational studies

|  | | Item No. | Recommendation | Page  No. | Relevant text from manuscript |
| --- | --- | --- | --- | --- | --- |
| Title and abstract | | 1 | (*a*) Indicate the study’s design with a commonly used term in the title or the abstract | 1,2 | A clinical severity scoring system for visceral leishmaniasis in immunocompetent patients in South Sudan.  A retrospective analysis was conducted of data from a cohort of 6,633 VL patients . |
|  |  |  | (*b*) Provide in the abstract an informative and balanced summary of what was done and what was found…. | 2 | Risk factors for death during treatment were identified using multivariable logistic regression models, and the regression coefficients were used to develop a severity scoring system. Sensitivity and specificity of score cut-offs were assessed by receiver operating characteristic (ROC) analysis.  **Results**  In multivariable models, risk factors for death in adult VL patients were: anaemia (odds ratio (OR) 4.46 (95% CI 1.58-12.6) for Hb <6g/dL compared with ≥9g/dL), nutritional status (OR 4.84 (2.09-11.2) for BMI <13 kg/m² compared with ≥16 kg/m²), weakness (OR 4.20 (1.82-9.73) for collapsed compared with normal weakness), jaundice (OR 3.41 (1.17-9.95)), and oedema/ascites (OR 4.86 (1.67-14.1)). For children and adolescents the risk factors were: age (OR 10.7 (6.3-18.3) for age <2 years compared with 6-18 years), anaemia (OR 7.76 (4.15-14.5) for Hb <6g/dL compared with ≥9g/dL), weakness (OR 3.13 (22.8-105.2) for collapsed compared with normal weakness), and jaundice (OR 12.8 (4.06-40.2)). Severity scoring predictive ability was 74.4% in adults and 83.4% in children and adolescents. |
| Introduction | | | | |  |
| Background/rationale | 2 | | Explain the scientific background and rationale for the investigation being reported | 2,3 | SSG is often poorly tolerated, and toxicity results in a significant incidence of serious adverse events such as pancreatic, hepato- and nephrotoxicity, cardiotoxicity, gastro-intestinal disorders and, in pregnant women, spontaneous abortion (8-11). For this reason, SSG is contraindicated for specific patient groups (e.g. pregnant women or HIV co-infected) or in patients with severe VL. These patients should be treated with liposomal amphotericin B (AmBisome) (12). AmBisome is much better tolerated but is much more expensive and requires cold chain transportation, cool storage, intravenous administration, and hospitalisation for at least 12 days. These are major challenges in the resource limited context of South Sudan, meaning that rational use of AmBisome is currently a key operational requirement. AmBisome needs to be reserved for severely ill VL patients who are at high risk of dying or at risk of SSG intolerability (12-14), whilst standard SSG/PM treatment can continue to be administered to patients with uncomplicated VL (7). In resource-limited settings, clinicians require a simple clinical tool to identify VL patients who are at increased risk of dying, and who need specialised treatment with liposomal amphotericin B and other supportive care. |
| Objectives | 3 | | State specific objectives, including any prespecified hypotheses | 3 | The aim of this study was to develop an evidenced-based risk scoring system which could be used as a clinical decision making tool in the field, to help clinicians decide whether a VL patient requires intensive VL care and treatment with AmBisome or standard VL management and less intensive monitoring. The risk scoring system would be based on risk factors for death during treatment in a retrospective cohort of VL patients, and would be validated internally and against older cohorts of VL patients treated by MSF in South Sudan. |
| Methods | | | | |  |
| Study design | | 4 | Present key elements of study design early in the paper | 3 | A retrospective analysis was conducted from a cohort of 6,831 VL patients. |
| Setting | | 5 | Describe the setting, locations, and relevant dates, including periods of recruitment, exposure, follow-up, and data collection | 3 | The analysis was conducted with routinely collected data from a cohort of 6,831 VL patients who attended the hospital of Médecins Sans Frontières (MSF) in Lankien, Jonglei state, South Sudan during the VL outbreak between July 2013 and June 2015 |
| Participants | | 6 | (*a*) *Cohort study*—Give the eligibility criteria, and the sources and methods of selection of participants. Describe methods of follow-up  *Case-control study*—Give the eligibility criteria, and the sources and methods of case ascertainment and control selection. Give the rationale for the choice of cases and controls  *Cross-sectional study*—Give the eligibility criteria, and the sources and methods of selection of participants | 3 | Patients with incomplete data and who defaulted from treatment were excluded. VL-HIV coinfected patients were excluded because treatments and outcomes are different in this small immunocompromised subgroup (during the study period the HIV/VL rate in Lankien was only 0.15%).  There is no systematic follow up of patients after discharge, because they return to areas remote from the treatment centre.. |
|  |  |  | (*b*) *Cohort study*—For matched studies, give matching criteria and number of exposed and unexposed  *Case-control study*—For matched studies, give matching criteria and the number of controls per case |  |  |
| Variables | | 7 | Clearly define all outcomes, exposures, predictors, potential confounders, and effect modifiers. Give diagnostic criteria, if applicable | 3,4 | Our analysis was based on a binary outcome of died (during or immediately after treatment) or survived treatment. “Survived” means that the patient was discharged after successful clinical response to treatment: absence of fever, reduction of spleen and liver size, increased haemoglobin, restored appetite, and feeling well. Patients with an increased risk of treatment failure or relapse (i.e. patients with a prior episode of VL, or patients with inadequate or doubtful clinical response) require a negative parasitological test-of-cure by aspirate microscopy to confirm cure of VL (…). A defaulter was defined as a patient who did not complete treatment, and had an unknown outcome.  VL was diagnosed if a patient presented with prolonged fever, wasting (BMI <16 kg/m2 or WHZ <-2) splenomegaly and/or lymphadenopathy, and a positive rapid rK39 diagnostic test (IT Leish/OptiLeish). If the rK39 test was negative but the patient presented with all of the clinical features of VL, a direct agglutination test (DAT) was performed. In a patient with clinical VL symptoms and a history of VL treatment, a lymph node or spleen aspirate was conducted (microscopic examination of tissue) to confirm a diagnosis of VL relapse (17). |
| Data sources/ measurement | | 8* | For each variable of interest, give sources of data and details of methods of assessment (measurement). Describe comparability of assessment methods if there is more than one group | 4 | age (years), sex (female/male), presence/absence of jaundice, lymphadenopathy, oedema/ascites; prior episode/relapse of VL, Hb level (g/dL), spleen size (cm below the left costal margin), self-reported duration of illness (months), and nutritional status (body mass index (BMI) in patients ≥19years (kg/m²) and weight for height Z scores (WHZ) in patients <19 years old). State of weakness, was defined as ‘normal weakness’ (non-severe); ‘severe weakness’ (if patient needs assistance in walking or, in babies, if unable to sit up); or ‘collapse’ (if patient is unable to sit or drink or, in babies, if hypotonic and unable drink unaided). All of these variables have been identified as risk factors for death in earlier VL patient cohorts from South Sudan (2, 5, 15, 16). |
| Bias | | 9 | Describe any efforts to address potential sources of bias | 4 | As the aim is to predict a patient’s prognosis at the time of diagnosis, the treatment was not included in the analysis. |
| Study size | | 10 | Explain how the study size was arrived at | 3, 6 | A retrospective analysis was conducted of routinely collected data from a cohort of 6,821 VL patients  After excluding patients with incomplete data (n=8), HIV co-infection (n=11), who defaulted from treatment (n=159) or referred to another non MSF facility (n=10) the total sample size was 6,633. |

Continued on next page

| Quantitative variables | | 11 | Explain how quantitative variables were handled in the analyses. If applicable, describe which groupings were chosen and why | | | 4 | All of these variables have been identified as risk factors for death in earlier VL patient cohorts from South Sudan (2, 5, 15, 16). | |  |
| --- | --- | --- | --- | --- | --- | --- | --- | --- | --- |
| Statistical methods | | 12 | (*a*) Describe all statistical methods, including those used to control for confounding | | | 4 | *Risk factors*  Univariate and multivariable logistic regression models were used to quantify the associations of risk factors with death as odds ratios (ORs) with 95% confidence intervals (CIs). Two prediction models were built: one with patients aged 19 years and older (adults), and one with the patients younger than 19 years (defined as children and adolescents). Variables were included in the multivariable analysis if they had a significance level of p <0.2 in the univariate analysis, and backwards elimination was then used to build final prediction models.  As the aim is to predict a patient’s prognosis at the time of diagnosis, the treatment should not be included. | |  |
|  |  |  | (*b*) Describe any methods used to examine subgroups and interactions | | |  | VL treatment effect was not analysed, because in observational studies indications for treatment are usually not standardised, and confounding by indication could lead to bias. Moreover, as the aim is to predict a patient’s prognosis at the time of diagnosis, the treatment should not be included. | |  |
|  |  |  | (*c*) Explain how missing data were addressed | | | 4 | Missing data (incomplete data ) were excluded from analyses | |  |
|  |  |  | (*d*) *Cohort study*—If applicable, explain how loss to follow-up was addressed  *Case-control study*—If applicable, explain how matching of cases and controls was addressed  *Cross-sectional study*—If applicable, describe analytical methods taking account of sampling strategy | | |  | N/A :There is no systematic follow up of patients after discharge, because they return to areas remote from the treatment centre. | |  |
|  |  |  | (*e*) Describe any sensitivity analyses | | | 5,6 | *Discriminative ability of the risk scoring system*  The discriminative (predictive) ability of the severity scoring system was assessed using receiver operating characteristic (ROC) analysis ([25](#_2bn6wsx), [26](#_qsh70q)). Discriminative ability was quantified by area under the curve (AUC), categorised as: ‘no better than a random guess’ (AUC 0.5-0.6); ‘poor’ (AUC 0.6-0.7); ‘fair’ (AUC 0.7-0.8); ‘good’ (AUC 0.8-0.9) or ‘excellent’ (AUC 0.9-1.0) (23, 24).  *Determination of severity score thresholds*  ROC analysis was used to calculate the sensitivity and specificity of different risk score thresholds (25, 26). The optimum threshold can be determined by considering the clinical and operational implications of the sensitivity and specificity of different thresholds.  *Validation*  The severity scoring system was validated in three datasets from previous studies in South Sudan: two from patients treated in Lankien during 1999-2002 (N=708) and during 2002-2005 (N=1882), and one from patients treated in Malakal during 2002-2005 (N= 1757) (2, 15). Validation was performed with the use of the Z test by comparing the predictive ability (AUC) of the severity scoring system across the datasets (27). | |  |
| Results | | | | | | | | |  |
| Participants | | 13* | (a) Report numbers of individuals at each stage of study—eg numbers potentially eligible, examined for eligibility, confirmed eligible, included in the study, completing follow-up, and analysed | | | 3, 6 | | A retrospective analysis was conducted of routinely collected data from a cohort of 6,821 VL patients. After excluding patients with incomplete data (n=8), HIV co-infection (n=11), who defaulted from treatment (n=159) or referred to another non MSF facility (n=10) the total sample size was 6,633.  Our analysis was based on a binary outcome of died (during or immediately after treatment) or survived treatment:  Mortality data was captured during the complete time of admission in the hospital, until discharge or death; the longest admission duration was 134 days. |  |
|  |  |  | (b) Give reasons for non-participation at each stage | | |  | | N/A |  |
|  |  |  | (c) Consider use of a flow diagram | | |  | |  |  |
| Descriptive data | | 14* | (a) Give characteristics of study participants (eg demographic, clinical, social) and information on exposures and potential confounders | | | 6, 9 | | The total sample size was 6,633. Of these, 3,631 (54.7%) were male and 3,002 (45.3%) were female. Out of the 6,614 patients of whom the duration of illness was known 6,087 (92%) presented within 1 months after onset of symptoms, and no patient presented later than 6 months. Of the 6,615 patients whose treatment regime was known, 5,149 patients were treated with SSG/PM and 1,466 patients with AmBisome. The data comprised 4,931 (74.3%) children and adolescents (aged < 19 years) and 1,702 (25.7%) adults (≥19 years). Mortality data was captured during the complete time of admission in the hospital, until discharge or death; the longest admission duration was 134 days. In total 6,447 patients (97.7%) survived and 186 (2.8%) died during treatment; 33% (49/186) of the deaths occurred within 48 hours of admission. The characteristics of patients in each age group who died compared with those who survived are shown in Table 1. Mortality in children and adolescents was 2.4% compared with 4.1% among adults (OR 1.78 (95% CI 1.32-2.41)). |  |
|  |  |  | (b) Indicate number of participants with missing data for each variable of interest | | | 7 | | Table 1. |  |
|  |  |  | (c) *Cohort study*—Summarise follow-up time (eg, average and total amount) | | | 3 | | There is no systematic follow up of patients after discharge, because they return to areas remote from the treatment centre. Our analysis was based on a binary outcome of died (during or immediately after treatment) or survived treatment. |  |
| Outcome data | | 15* | *Cohort study*—Report numbers of outcome events or summary measures over time | | | 6 | | The sample size with a known outcome as 6,633 :  Mortality data was captured during the complete time of admission in the hospital, until discharge or death; the longest admission duration was 134 days. In total 6,447 patients (97.7%) survived and 186 (2.8%) |  |
|  |  |  | *Case-control study—*Report numbers in each exposure category, or summary measures of exposure | | |  | |  |  |
|  |  |  | *Cross-sectional study—*Report numbers of outcome events or summary measures | | |  | |  |  |
| Main results | | 16 | (*a*) Give unadjusted estimates and, if applicable, confounder-adjusted estimates and their precision (eg, 95% confidence interval). Make clear which confounders were adjusted for and why they were included | | | 7, 8,  10, 11 | | A prediction model was built therefore we included all the variables.  Table 2 and table 3  *Risk factors in adults*  Univariate analysis showed that age, Hb, state of weakness, nutritional status, jaundice, and oedema/ascites were strongly associated with VL mortality (Table 2): patients >45 years old had 3.4-fold higher odds of death (OR 3.42 (95% CI 1.64-7.16)) compared with patients aged 18-25 years; patients in a state of collapse were 8 times more likely to die (OR 7.80 (95% CI 3.66-16.6)) compared with patients who arrived in a ‘normal’ state of weakness; Hb levels <6g/dL increased the odds of dying almost 9-fold (OR 8.67 (95% CI 3.32-22.6)) compared with levels ≥9g/dL; and BMI <13 kg/m2 was associated with 7.6-fold higher odds of death (OR 7.56 (95% CI 3.45-16.6)) compared with BMI ≥ 16 kg/m2. Presence of jaundice and oedema/ascites increased the odds of dying 8-fold (OR 7.95 (95% CI 3.07-20.6)) and almost 7-fold (OR 6.87 (95% CI 2.69-17.5)), respectively. Sex, lymphadenopathy, prior episode VL, duration of illness, and spleen size were not associated with risk of death.  The effects of Hb, state of weakness, nutritional status, jaundice, and oedema/ascites were reduced by mutual adjustment, and age was eliminated from the final prediction model (Table 2). In this model, patients in a state of collapse were 4 times more likely to die (OR 4.20 (95% CI 1.82-9.73)) compared with patients who arrived in a ‘normal’ state of weakness; Hb levels <6g/dL increased the odds of dying 4.5-fold (OR 4.46 (95% CI 1.58-12.6)) compared with levels ≥9g/dL; and BMI <13 kg/m2 was associated with almost 5-fold higher odds of death (OR 4.84 (95% CI 2.09-11.2)) compared with BMI ≥ 16 kg/m2. Presence of jaundice and oedema/ascites increased the odds of dying >3-fold (OR 3.41 (95% CI 1.17-9.95)) and almost 5-fold (OR 4.86 (95% CI 1.67-14.1)), respectively.  *Risk factors in children and adolescents*  The univariate analyses of children and adolescents, showed that age, Hb, state of weakness, jaundice, oedema/ascites and WHZ were strongly associated with VL mortality (Table 3). Patients < 2 years old had >17-fold higher odds of death (OR 17.3 (95% CI 10.5-28.6)) compared with patients aged 6-18 years; patients in a state of collapse were 39 times more likely to die (OR 39.3 (95% CI 12.2-126.3)) compared with patients who arrived in a ‘normal’ state of weakness; Hb levels <6g/dL increased the odds of dying 19-fold (OR 19.0 (95% CI 10.8-33.4)) compared with levels ≥9g/dL; and WHZ <-4 was associated with almost 3-fold higher odds of death (OR 2.74 (95% CI 1.58-4.90)) compared with WHZ > -2. Presence of jaundice and oedema/ascites increased the odds of dying >9-fold (OR 9.38 (95% CI 3.50-25.2)) and >5-fold (OR 5.31 (95% CI 2.25-12.7)), respectively. For patients with lymphadenopathy, the odds of dying reduced by half. (OR 0.49 (95% CI 0.33-0.73) Sex, prior episode VL, and spleen size were not associated with risk of death. Duration of illness did not have sufficient cases for analyses.  The effects of age, Hb, state of weakness and jaundice were reduced by mutual adjustment, and lymphadenopathy, nutritional status and oedema/ascites were eliminated from the final prediction model (Table 3). In this model, patients < 2 years old had almost 11-fold higher odds of death (OR 10.7 (95% CI 6.31-18.26)) compared with patients aged 6-18 years; patients in a state of collapse were almost 23 times more likely to die (OR 22.8 (95% CI 4.95-105.2)) compared with patients who arrived in a ‘normal’ state of weakness; Hb levels <6g/dL increased the odds of dying nearly 8-fold (OR 7.76 (95% CI 4.15-14.5)) compared with levels ≥9g/dL; and presence of jaundice increased the odds of dying 3.4-fold (OR 3.41 (95% CI 1.17-9.95)). |  |
|  |  |  | (*b*) Report category boundaries when continuous variables were categorized | | | 9 | | Table 1 |  |
|  |  |  | (*c*) If relevant, consider translating estimates of relative risk into absolute risk for a meaningful time period | | |  | |  |  |
| Other analyses | | 17 | | Report other analyses done—eg analyses of subgroups and interactions, and sensitivity analyses | | 12, 13, 14 | | *Severity scoring system*  The scores for each variable in the final prediction model are shown in Table 4, and the range of probabilities for each score (due to rounding up or down of regression coefficients) are presented in Table 5. For adults, probability of death exceeded 10% for risk scores ≥3 (6.1% (104/1695) of adults); in children and adolescents, the threshold for exceeding a 10% probability of death was a score ≥6 (6.8% (339/4921) of children).  For the severity scoring of the adults the classification matrix resulted in 80.8% correctly predicted deaths. The AUC of the severity scores of the adults gave an overall predictive performance of 74.4% (95 CI 68.0%-81.0%), indicating ‘fair’ predictive ability (Figure 1). For children and adolescents, the classification matrix showed 80.6% correctly predicted. The AUC was 83.4% (95% CI 78.0%-86.8), interpreted as ‘good’ predictive ability (Figure 1). Sensitivity >55% required a score ≥2 in adults (sensitivity 57%, specificity 82%) and ≥5 in children and adolescents (sensitivity 64%, specificity 91%). Sensitivity >75% required a score ≥1 in adults (sensitivity 86%, specificity 44%) and ≥4 in children and adolescents (sensitivity 75%, specificity 81%) (Table 6).  *Validation*  External validation of the scoring for adults with the Lankien datasets of 1999-2002, 2002-2005 and the Malakal dataset of 2002-2005 yielded AUC of 72.2%, 79.5% and 71.2%, respectively. Discriminative ability did not differ significantly across the four datasets (p= 0.48). For children and adolescents, corresponding AUC were 72.2%, 82.8% and 76.6%, with only very weak evidence of a difference in discriminative ability across the three datasets (p = 0.13). | |
| Discussion | | | | | | | | |  |
| Key results | 18 | | | Summarise key results with reference to study objectives | | 15 | | In this study, the risk factors for death in VL patients in South Sudan during the VL epidemic from 2013 to 2015 were analysed. Significant risk factors for adult patients were nutritional status (BMI), Hb, weakness status, jaundice and oedema/ascites. In children and adolescents, the risk factors were age, Hb, weakness status and jaundice. Using these risk factors, an evidence-based clinical severity scoring system was developed that may be able to determine reliably and easily a patient’s risk of dying, thereby enhancing the rational use of more costly and complex VL treatments. The overall accurate predictive ability of this severity scoring system was confirmed by external validation in other data from the same setting. |  |
| Limitations | 19 | | | Discuss limitations of the study, taking into account sources of potential bias or imprecision. Discuss both direction and magnitude of any potential bias | | 15, 16 | | On the other hand, older age (>45 years), which was strongly associated with mortality in the univariate analysis, was not retained as an independent risk factor in the multivariable regression model, contrary to earlier studies (15, 16, 22).  In these earlier studies, most elderly patients were treated with SSG, whereas in the present study 87% of the patients older than 45 years were treated with AmBisome (15, 16, 28). Several studies had demonstrated a high mortality in older patients due to SSG toxicity, and during those earlier studies there was no or only limited AmBisome available (12). Therefore in our dataset, AmBisome may have mediated the effect of older age on VL mortality. This seems to confirm the recommendation that treatment with AmBisome may be lifesaving for the elderly VL patients (9).    In contrast with two previous studies we did not find that splenomegaly was associated with increased risk of death (15, 29). Conversely, we found a crude inverse association of lymphadenopathy with risk of death (although not evident when adjusted for other risk factors). We can think of no plausible explanation for these apparently anomalous findings.  The study was based on a cohort of South Sudanese patients, and therefore the predictors of death and the severity scoring system should not be generalised to patient populations in other countries (..)  Another limitation is that this retrospective study was conducted with routinely collected programme data. Therefore it may be missing out on other important risk factors that were not included in this database, such as laboratory parameters.  Although one third of deaths occurred within the first 48 hours after admission, which may limit the impact of our severity scoring system on mortality, the predictive ability and simplicity of the system means that it can be easily operationalised and implemented in the field. |  |
| Interpretation | 20 | | | Give a cautious overall interpretation of results considering objectives, limitations, multiplicity of analyses, results from similar studies, and other relevant evidence | | 16 | | The scoring system presented in this study only includes clinical parameters and a simple Hb lab test, and it therefore presents a practical tool that can be used in all field hospitals and health centres in the VL endemic areas in South Sudan. Clinicians will use their clinical judgement and experience to make treatment decisions, aided by the risk scoring tool. Given the strong associations between known risk factors and mortality, it would not be ethical to attempt a randomised trial of the severity scoring system, but we would hope to see a (continued) overall improvement in treatment outcomes in VL programmes in South Sudan until new safe, effective, and affordable treatment becomes available for all patients. |  |
| Generalisability | 21 | | | Discuss the generalisability (external validity) of the study results | | 16 | | The study was based on a cohort of South Sudanese patients, and therefore the predictors of death and the severity scoring system should not be generalised to patient populations in other countries. In particular, where VL is caused by other Leishmania strains, where HIV co-infection is more prevalent, or where resistance to SSG is more common or AmBisome is more affordable. |  |
| Other information | | | |  | | | | |  |
| Funding | 22 | | | Give the source of funding and the role of the funders for the present study and, if applicable, for the original study on which the present article is based |  | | | N/A. |  |

*Give information separately for cases and controls in case-control studies and, if applicable, for exposed and unexposed groups in cohort and cross-sectional studies.

**Note:** An Explanation and Elaboration article discusses each checklist item and gives methodological background and published examples of transparent reporting. The STROBE checklist is best used in conjunction with this article (freely available on the Web sites of PLoS Medicine at http://www.plosmedicine.org/, Annals of Internal Medicine at http://www.annals.org/, and Epidemiology at http://www.epidem.com/). Information on the STROBE Initiative is available at www.strobe-statement.org.
