# Supplementary figures and images for: A clinical severity scoring system for visceral leishmaniasis in immunocompetent patients in South Sudan
Source: PLoS Negl Trop Dis. 2017 Oct 2;11(10):e0005921. doi: 10.1371/journal.pntd.0005921 (PMC5638606; doi:10.1371/journal.pntd.0005921)

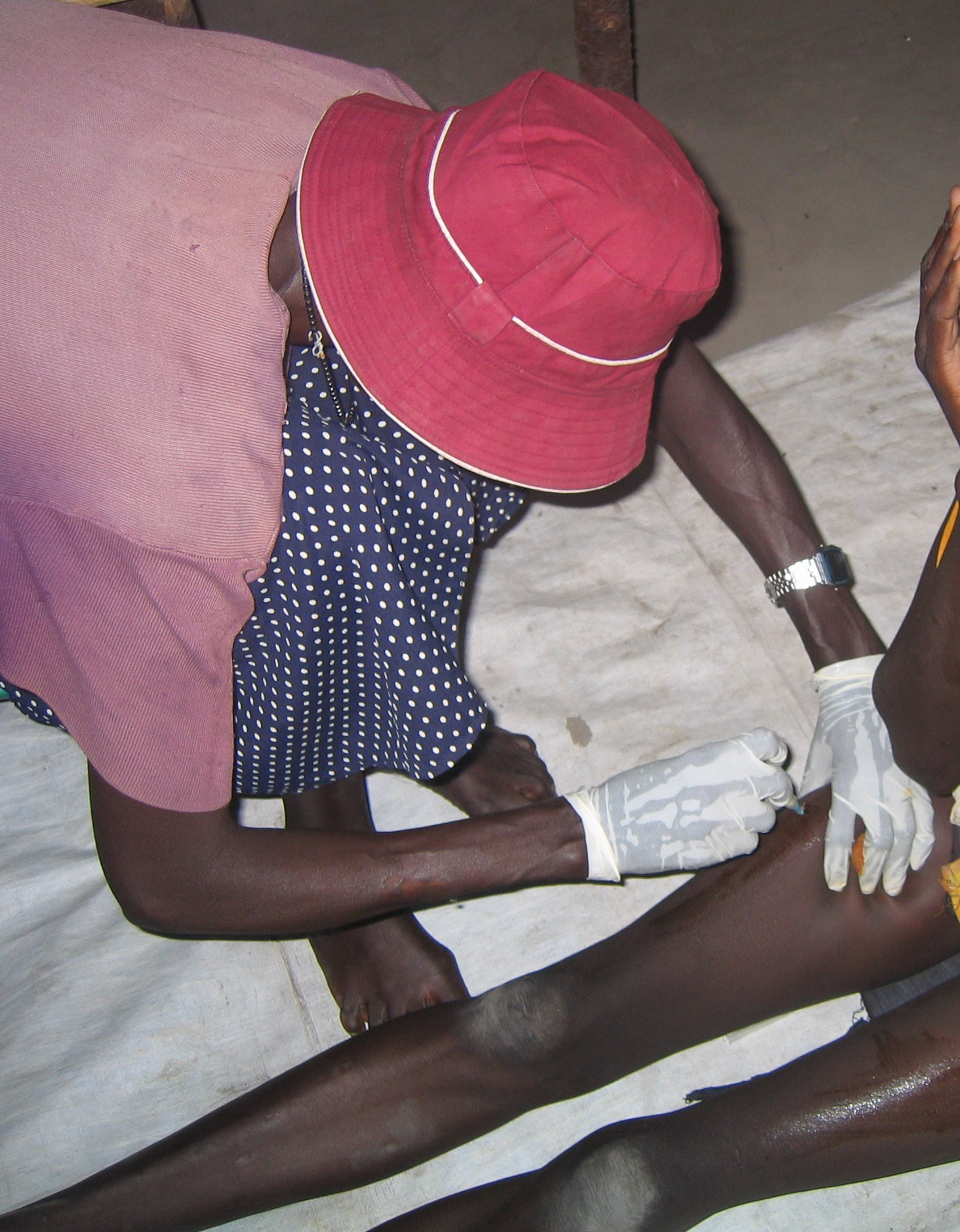

Supplement: S1 Fig — Image credit: M.den Boer, MSF. (TIF) [file pntd.0005921.s002.tif]
